# Development of PD-1/PD-L1 Pathway in Tumor Immune Microenvironment and Treatment for Non-Small Cell Lung Cancer

**DOI:** 10.1038/srep13110

**Published:** 2015-08-17

**Authors:** Jiabei He, Ying Hu, Mingming Hu, Baolan Li

**Affiliations:** 1 General department Beijing Chest Hospital, Capital Medical University/Beijing Tuberculosis and Thoracic Tumor Research Institute, Tongzhou District, Beijing, PR China

## Abstract

Lung cancer is currently the leading cause of cancer-related death in worldwide, non-small cell lung cancer (NSCLC) accounts for about 85% of all lung cancers. Surgery, platinum-based chemotherapy, molecular targeted agents and radiotherapy are the main treatment of NSCLC. With the strategies of treatment constantly improving, the prognosis of NSCLC patients is not as good as before, new sort of treatments are needed to be exploited. Programmed death 1 (PD-1) and its ligand PD-L1 play a key role in tumor immune escape and the formation of tumor microenvironment, closely related with tumor generation and development. Blockading the PD-1/PD-L1 pathway could reverse the tumor microenvironment and enhance the endogenous antitumor immune responses. Utilizing the PD-1 and/or PD-L1 inhibitors has shown benefits in clinical trials of NSCLC. In this review, we discuss the basic principle of PD-1/PD-L1 pathway and its role in the tumorigenesis and development of NSCLC. The clinical development of PD-1/PD-L1 pathway inhibitors and the main problems in the present studies and the research direction in the future will also be discussed.

Lung cancer is currently the leading cause of cancer-related death in the worldwide. In China, the incidence and mortality of lung cancer is 5.357/10000, 4.557/10000 respectively, with nearly 600,000 new cases every year^1^. Non-small cell lung cancer (NSCLC) accounts for about 85% of all lung cancers, the early symptoms of patients with NSCLC are not very obvious, especially the peripheral lung cancer. Though the development of clinic diagnostic techniques, the majority of patients with NSCLC have been at advanced stage already as they are diagnosed. Surgery is the standard treatment in the early stages of NSCLC, for the advanced NSCLC, the first-line therapy is platinum-based chemotherapy. In recent years, patients with specific mutations may effectively be treated with molecular targeted agents initially. The prognosis of NSCLC patients is still not optimistic even though the projects of chemotherapy as well as radiotherapy are continuously ameliorating and the launch of new molecular targeted agents is never suspended, the five-year survival rate of NSCLC patients is barely more than 15%^2^, the new treatment is needed to be opened up.

During the last few decades, significant efforts of the interaction between immune system and immunotherapy to NSCLC have been acquired. Recent data have indicated that the lack of immunologic control is recognized as a hallmark of cancer currently. Programmed death-1 (PD-1) and its ligand PD-L1 play a key role in tumor immune escape and the formation of tumor microenvironment, closely related with tumor generation and development. Blockading the PD-1/PD-L1 pathway could reverse the tumor microenvironment and enhance the endogenous antitumor immune responses.

In this review, we will discuss the PD-1/PD-L1 pathway from the following aspects: the basic principle of PD-1/PD-L1 pathway and its role in the tumorigenesis and development of NSCLC, the clinical development of several anti-PD-1 and anti-PD-L1 drugs, including efficacy, toxicity, and application as single agent, or in combination with other therapies, the main problems in the present studies and the research direction in the future.

## Immune checkpoint pathways and cancer

Cancer as a chronic, polygene and often inflammation-provoking disease, the mechanism of its emergence and progression is very complicated. There are many factors which impacted the development of the disease, such as: environmental factors, living habits, genetic mutations, dysfunction of the immune system and so on. At present, increasing evidence has revealed that the development and progression of tumor are accompanied by the formation of special tumor immune microenvironment. Tumor cells can escape the immune surveillance and disrupt immune checkpoint of host in several methods, therefore, to avoid the elimination from the host immune system. Human cancers contain a number of genetic and epigenetic changes, which can produce neoantigens that are potentially recognizable by the immune system^3^, thus trigger the body’s T cells immune response. The T cells of immune system recognize cancer cells as abnormal primarily, generate a population of cytotoxic T lymphocytes (CTLs) that can traffic to and infiltrate cancers wherever they reside, and specifically bind to and then kill cancer cells. Effective protective immunity against cancer depends on the coordination of CTLs^4^. Under normal physiological conditions, there is a balance status in the immune checkpoint molecule which makes the immune response of T cells keep a proper intensity and scope in order to minimize the damage to the surrounding normal tissue and avoid autoimmune reaction. However, numerous pathways are utilized by cancers to up-regulate the negative signals through cell surface molecules, thus inhibit T-cell activation or induce apoptosis and promote the progression and metastasis of cancers^5^. Increasing experiments and clinical trails show that immunotherapeutic approaches utilizing antagonistic antibodies to block checkpoint pathways, can release cancer inhibition and facilitate antitumor activity, so as to achieve the purpose of treating cancer.

The present research of immune checkpoint molecules are mainly focus on cytotoxic T lymphocyte-associated antigen 4 (CLTA-4), Programmed death-1 (PD-1) and its ligands PD-L1 (B7H1) and PD-L2 (B7-DC). CTLA-4 regulates T cell activity in the early stage predominantly, and PD-1 mainly limits the activity of T-cell in the tumor microenvironment at later stage of tumor growth^6^. Utilizing the immune checkpoint blockers to block the interactions between PD-1 and its ligands has shown benefits in clinical trials, including the NSCLC patients. PD-1 and its ligands have been rapidly established as the currently most important breakthrough targets in the development of effective immunotherapy.

## The biological characteristics of PD-1/PD-L1 pathway and its role in tumor

### PD-1/PD-L1 pathway and its expression, regulation

PD-1 is a type 1 trans-membrane protein that encoded by the PDCD1 gene^7^. It is a member of the extended CD28/CTLA-4 immunoglobulin family and one of the most important inhibitory co-receptors expressed by T cells. The structure of the PD-1 includes an extracellular IgV domain, a hydrophobic trans-membrane region and an intracellular domain. The intracellular tail includes separate potential phosphorylation sites that are located in the immune receptor tyrosine-based inhibitory motif (ITIM) and in the immunoreceptor tyrosine-based switch motif (ITSM). Mutagenetic researches indicated that the activated ITSM is essential for the PD-1 inhibitory effect on T cells^8^. PD-1 is expressed on T cells, B cells, monocytes, natural killer cells, dendritic cells and many tumor-infiltrating lymphocytes (TILs)^9^. In addition, the research of Francisoet *et al.* showed that PD-1 was also expressed on regulatory T cells (Treg) and able to facilitate the proliferation of Treg and restrain immune response^10^.

PD-1 has two ligands: PD-L1 (also named B7-H1; CD274) and PD-L2 (B7-DC; CD273), that are both coinhibitory. PD-L1 is expressed on resting T cells, B cells, dendritic cells, macrophage, vascular endothelial cells and pancreatic islet cells. PD-L2 expression is seen on macrophages and dendritic cells alone and is far less prevalent than PD-L1 across tumor types. It shows much more restricted expression because of its more restricted tissue distribution. Differences in expression patterns suggest distinct functions in immune regulation across distinct cell types. The restricted expression of PD-L2, largely to antigen-presenting cells, is consistent with a role in regulating T-cell priming or polarization, whereas broad distribution of PD-L1 suggests a more general role in protecting peripheral tissues from excessive inflammation.

PD-L1 is expressed in various types of cancers, especially in NSCLC^11^,^12^, melanoma, renal cell carcinoma, gastric cancer, hepatocellular as well as cutaneous and various leukemias, multiple myeloma and so on^13^,^14^,^15^. It is present in the cytoplasm and plasma membrane of cancer cells, but not all cancers or all cells within a cancer express PD-L1^16^,^17^. The expression of PD-L1 is induced by multiple proinflammatory molecules, including types I and II IFN-γ, TNF-α, LPS, GM-CSF and VEGF, as well as the cytokines IL-10 and IL-4, with IFN-γ being the most potent inducer^18^,^19^. IFN-γ and TNF-α are produced by activated type 1 T cells, and GM-CSF and VEGF are produced by a variety of cancer stromal cells, the tumor microenvironment upregulates PD-L1 expression, thereby, promotes immune suppression. This latter effect is called “adaptive immune resistance”, because the tumor protects itself by inducing PD-L1 in response to IFN-γ produced by activated T cells^17^. PD-L1 is regulated by oncogenes, also known as the inherent immune resistance. PD-L1 expression is suppressed by the tumor suppressor gene: PTEN (phosphatase and tension homolog deleted on chromosome ten) gene. Cancer cells frequently contain mutated PTEN, which can activate the S6K1 gene, thus results in PD-L1 mRNA to polysomes increase greatly^20^, hence increases the translation of PD-L1 mRNA and plasma membrane expression of PD-L1. Parsa *et al.*’s research also demonstrated that neuroglioma with PTEN gene deletion regulate PD-L1 expression at the translational level by activating the PI3K/AKT downstream mTOR-S6K1signal pathway and, hence increase the PD-L1 expression^21^. Micro-RNAs also translationally regulate PD-L1 expression. MiRNA-513 is complementary to the 3′ untranslated region of PD-L1 and prevents PD-L1 mRNA translation^22^. In addition, a later literature reported that in the model of melanoma, the up-regulation of PD-L1 is closely related to the CD8 T cell, independent of regulation by oncogenes^13^. Noteworthily, the PD-L1 can bind to T cell expressed CD80, and at this point CD80 is a receptor instead of ligand to transmit negative regulated signals^23^.

### PD-1/PD-L1 mediate immune suppression by multiple mechanisms

Like the CTLA-4, the PD-1/PD-L1 pathway down-modulates T-cell response by regulating overlapping signal proteins in the immune checkpoint pathway. However, their functions are slightly different^24^. The CTLA-4 focuses on regulating the activation of T cells, while PD-1 regulates effector T-cell activity in peripheral tissues in response to infection or tumor progression^25^. Tregs that high-level expression of PD-1 have been shown to have immune inhibitory activity, thus, they are important for maintaining self-tolerance. In normal human bodies, this is a crucial step to protect against tissue damage when the immune system is activated in response to infection^26^. However, in response to immune attack, cancer cells overexpress PD-L1 and PD-L2. They bind to PD-1 receptor on T cells, inhibiting the activation of T-cells, thus suppressing T-cell attack and inducing tumor immune escape. Thus tumor cells effectively form a suitable tumor microenvironment and continue to proliferate^27^. PD-1/PD-L1 pathway regulates immune suppression by multiple mechanisms, specific performance of the following: ① Induce apoptosis of activated T cells: PD-1 reduces T cell survival by impacting apoptotic genes. During T cell activation, CD28 ligation sustains T cell survival by driving expression of the antiapoptotic gene Bcl-xL. PD-1 prevents Bcl-xL expression by inhibiting PI3K activation, which is essential for upregulation of Bcl-xL. Early studies demonstrated that PD-L1+ murine and human tumor cells induce apoptosis of activated T cells and that antibody blocking of PD-L1 can decrease the apoptosis of T cells and facilitate antitumor immunity^28^,^16^. ② Facilitate T cell anergy and exhaustion: A research shown that the occurrence of tumor is associated with chronic infection^29^. According to the study of chronic infection, PD-1 overexpressed on the function exhausted T cells, blocking the PD-1/PD-L1 pathway can restore the proliferation, secretion and cytotoxicity^30^. In addition, later research demonstrated that the exhaustion of TILs in the tumor microenvironment is closely related to the PD-L1 expression of tumor cells, myeloid cells derived from tumor^31^. ③ Enhance the function of regulatory T cells: PD-L1 can promote the generation of induced Tregs by down-regulating the mTOR, AKT, S6 and the phosphorylation of ERK2 and increasing PTEN, thus restrain the activity of effector T-cell^32^. Blocking the PD-1/PD-L1 pathway can increase the function of effector CD8 T-cell and inhibt the function of Tregs, bone marrow derived inhibition cells, thus enhance the anti-tumor response. ④ Inhibit the proliferation of T cells: PD-1 ligation also prevents phosphorylation of PKC-theta, which is essential for IL-2 production^33^, and arrests T cells in the G1 phase, blocking proliferation. PD-1 mediates this effect by activating Smad3, a factor that arrests cycling^34^. ⑤ Restrain impaired T cell activation and IL-2 production: PD-1/PD-L1 blocks the downstream signaling events triggered by Ag/MHC engagement of the TCR and co-stimulation through CD28, resulting in impaired T cell activation and IL-2 production. Signaling through the TCR requires phosphorylation of the tyrosine kinase ZAP70. PD-1 engagement reduces the phosphorylation of ZAP70 and, hence, inhibits downstream signaling events. In addition, signaling through PD-1 also prevents the conversion of functional CD8^+^ T effector memory cells into CD8^+^ central memory cells^35^ and, thus, reduces long-term immune memory that might protect against future metastatic disease. PD-L1 also promotes tumor progression by reversing signaling through CD80 into T cells. CD80-PD-L1 interactions restrain self-reactive T cells in an autoimmune setting^36^, therefore, their inhibition may facilitate antitumor immunity.

Researches on the mechanism of PD-1/PD-L1 pathway mediating immune escape are still ongoing, especially the mechanism of PD-L2 is still unclear. These researches provide the theoretical basis and research direction for the further immunotherapy targets research.

## The clinical significance of PD-1/PD-L1 pathway in NSCLC

Historically, immunotherapy has been ineffective in cases of NSCLC, which has been thought to be a type of non-immunogenic cancer; nevertheless, NSCLC can evade the immune system through various complex mechanisms which we have discussed above. Researchers had discovered that the count of peripheral and tumor lymphocytes which are capable of suppressing tumor immune surveillance reduced in patients with advanced non-small cell lung cancer^37^.

Some retrospective studies showed that the overexpression of PD-L1 and is closely related to the poor prognosis and high invasiveness in NSCLC patients^38^,^39^. Similar conclusions have reported in the other tumors such as hepatocarcinoma^40^, colorectal cancer. However, Yang *et al.*’s research demonstrated that, PD-L1 had higher positive results in lung adenocarcinoma with higher grade differentiation and vascular invasion and positive PD-L1 expression correlated with less cancer recurrence. PD-L1 expression is correlated with better relapse-free survival (RFS), but unrelated with overall survival (OS)^41^. The contradiction in the results of various researches may be caused by a number of different factors on PD-L1 expression itself. Such as the species selection, pathological types of tumors, the quality of the samples and material acquired method, test reagents and method, counting method and so on, all the factors mentioned above may have a significant impact on PD-L1 expression. Therefore, whether the intensity of PD-L1 expression could be used to predict the prognostic of NSCLC patients is controversial, it still needs further research.

In addition, a study involving 583 NSCLC cases with surgically resected for single-nucleotide polymorphism (SNP) analyses has reported that: there were no significant differences in different PD-1 SNP statuses (AA, GA, GG) and the ratio was extremely similar to the healthy control in a previous study. But the survival time of the patients with the GG phenotype of PD-1 was significantly shorter compared to the patients with GA or AA, and the GG phenotype patients significantly had a worse prognosis in the SCC population^42^.

The process of tumorigenesis, progression and metastasis is a process of complex and consecutive changes. The activation of oncogenes and the inactivation of tumor suppressor genes by mutations and the imbalance of immune system are all essential components of the process. An imbalance to the regulation of the immune system changes the tumor-specific T-cell immunity in the tumor microenvironment and adjusts the tumor progression and metastasis. It has been reported that the mutations of tumor-related genes are associated with the imbalance of immune system. In the process of the development of lung cancer, there are a lot of genetic mutations, such as EGFR, ALK and so on. Studies have shown that, PD-L1 expression in EGFR mutation samples is higher than in wild type, and the mutation can promote the expression of PD-L1^43^. It provides the theoretical basis for clinical application of the combination therapy of EGFR and PD-L1 inhibitor. But there is a study reported that, PD-L1 expression was not associated with the major driver mutation of lung adenocarcinoma in East Asians (EGFR, ALK, KRAS, BRAF)^42^. More detailed researches and large samples are still needed in this respect.

Researchers have shown that the PD-L1expression of tumors may be associated with the efficacy of NSCLC patients who received immunotherapy. Recently, a multicenter phase I clinical trial result of pembrolizumab used for treatment of advanced NSCLC patients was published in New England Journal of Medicine. The result suggested that the response rate for patients with PD-L1 positive (a proportion score of at least 50%) exceeded the PD-L1 negative (a score of 1 to 49% and the group with a score of less than 1%) for previously treated patients (P < 0.001) and for previously untreated patients (P = 0.01). Progression-free and overall survival were shorter among patients with a proportion score of 1 to 49% or a score of less than 1% than among those with a score of at least 50%^44^.

In a multi-center clinical trail focused on specific antibody nivolumab that specifically blocking PD-1 showed that cumulative response rates (all doses) were 18% among patients with NSCLC (14 of 76 patients). Immunohistochemical analysis was performed on pretreatment tumor specimens obtained from 42 patients, objective response rate of PD-L1 positive and negative was 9 of 25 patients (36%), 0% (0/17) (P = 0.006)respectively^45^. However, PD-L1 negative patients may also benefit from anti-PD-1/PD-L1treatment which has also been reported by a few of clinical trails^46^. Thus, some potential patients who can benefit from anti-PD-1/PD-L1treatment may be eliminated if PD-L1 positive is deemed as screening standard.

Currently, studies focused on the reasons of non-response to anti-PD-1/PD-L1 therapy for patients with NSCLC scarely have reported. However, analyzing on the basis of currently available clinical and basic studies, it can be assumed that different responses to anti-PD-1/PD-L1 therapy are not caused by single factors, but the result of the interaction of various factors (different PD-1 SNP statuses, genetic mutations , PD-L1 expression, histopathologic types of NSCLC and so on).

In addition, some investigators found that PD-1 expression on tumor cells^47^. This finding could have therapeutic implications and further studies are warranted to confirm these findings.

Therefore, the mechanism and the relationship during PD-1/PD-L1 and the immune therapy efficacy and the selection of patients still need further basic studies and large sample phase II or III clinical trials to be confirmed.

## Drugs targeting PD-1, PD-L1 and combination with other cancer treatments

### Anti-PD-1 antibodies

#### Nivolumab

Nivolumab (BMS-936558, Brand name: Opdivo) is a human monoclonal IgG4 antibody that essentially lacks detectable antibody-dependent cellular cytotoxicity (ADCC). Inhibition by monoclonal antibody of PD-1 on CD8+ TILs within lung cancers can restore cytokine secretion and T-cell proliferation^48^. Results of a larger phase I study in 296 patients (236 patients evaluated) reported that the objective response (complete or partial responses) of patients with NSCLC was 18%. A total of 65% of responders had durable responses lasting for more than 1 year. Stable disease lasting 24 weeks was seen in patients with NSCLC. PD-L1expression was tested in 42 patients: 9 of 25(36%) patients whose PD-L1 expression positive were objectively response to PD-1 blockade treatment, while the remaining 17 nonresponsive patients were negative^45^.

In another early phase I trial of nivolumab^49^, an objective response was observed in 22 patients (17%; 95% CI, 11%–25%) in a dose-expansion cohort of 129 previously treated patients with advanced NSCLC. Six additional patients who had an unconventional immune-related response were not included. Moreover, the median duration of response was exceptional for 17 months. Although the median PFS in the cohort was 2.3 months and the median overall survival was 9.9 months, it seemed clear that those who responded had sustained benefit. Specifically, the 2-year overall survival rate was 24%, and many remained in remission after completing 96 weeks of continuous therapy.

Single-agent trials of nivolumab are planning or ongoing on NSCLC (NCT01721759, NCT02066636). In addition, there are clinical randomized trials which focus on the comparison of nivolumab and plain-based combination chemotherapy (NCT02041533, NCT01673867). In March 4, 2015, nivolumab was approved by the US Food and Drug Administration for treatment of patients with metastatic NSCLC (squamous cell carcinoma), when progression of their diseases during or after chemotherapy with platinum-based drugs.

#### Pembrolizumab

Pembrolizumab (MK-3475) is a highly selective, humanized monoclonal antibody with activity against PD-1 that contains a mutation at C228P designed to prevent Fc-mediated ADCC. It is now in the clinical research phases for patients with advanced solid tumors. Its safety and efficacy were evaluated in a phase I clinical trial of KEYNOTE-001. The best response according of 38 cases of patients which initially accepted pembrolizumab 10 mg/kg q3wwas 21% (based on RECIST1.1 evaluation) and the median PFS of responder still has not reached until 62 weeks. The researchers also found that the antitumor activity of pembrolizumab was associated with the PD-L1expression^44^,^50^. The critical values of the expression of PD-L1 will be validated in 300 cases of patients which will soon been rolled into the study.

Clinical trial of pembrolizumab monotherapy is ongoing for patients with NSCLC (NCT01840579). Randomized trials comparing pembrolizumab to combination chemotherapy (NCT02142738) or single-agent docetaxel (NCT01905657) are ongoing in PD-L1 positive patients with NSCLC.

#### Pidilizumab (CT-011)

Pidilizumab is a humanized IgG-1K recombinant anti-PD-1 monoclonal antibody that has demonstrated antitumor activity in mouse cancer models. In a first-in-human phase I dose-escalation study in patients with only advanced hematologic cancers, there is no clinical trials of NSCLC presently^51^.

### Anti-PD-L1 antibodies

Another therapeutic method based on the PD-1/PD-L1 pathway is by specific binding between antibody and PD-L1, thus preventing its activity. It was speculated that utilizing PD-L1 as therapeutic target maybe accompanied by less toxicity in part by modulating the immune response selectively in the tumor microenvironment. However, since PD-L2 expressed by tumor cells or some other tumor-associated molecules may play a role in mediating PD-1-expressing lymphocytes, it is conceivable that the magnitude of the anti-tumor immune response could also be blunted.

#### BMS-936559

BMS-936559/MDX1105 is a fully humanized, high affinity, IgG4 monoclonal antibody that react specifically with PD-L1, thus inhibiting the binding of PD-L1 and PD-1, CD80 (which binds not only PD-L1 but also CTLA-4 and CD28). Initial results from a multicenter and dose-escalation phase I trial of 207 patients(including 75 cases of patients with NSCLC) showed durable tumor regression (objective response rate of 6%–17%) and prolonged stabilization of disease (12%–41% at 24weeks) in patients with advanced cancers, including NSCLC, melanoma and kidney cancer. In patients with NSCLC, there were five objective responses (in 4 patients with the nonsquamous subtype and 1 with the squamous subtype) at doses of 3 mg/kg and 10 mg/kg, with response rates of 8% and 16%, respectively. Six additional patients with NSCLC had stable disease lasting at least 24 weeks^52^.

#### MPDL3280A

MPDL3280A is a human IgG1 antibody that targets PD-L1. Its Fc component has been engineered to not activate antibody-dependent cell cytotoxicity. In a recently reported phase I study, a 21% response rate was noted in patients with metastatic melanoma, RCC or NSCLC^53^, including several patients who demonstrated shrinkage of tumor within a few days of initiating treatment.

Fifty-two patients were enrolled in an expansion cohort of the phase I trial of MPDL3280A, 62% of them were heavily pretreated NSCLC (≥3 lines of systemic therapy) and the ORR was 22%^54^. Analysis of biomarker data from archival tumor samples demonstrated a correlation between PD-L1 status and response and lack of progressive disease^55^.

#### MEDI4736

MEDI4736 is a human IgG1 antibody that binds specifically to PD-L1, thus preventing PD-L1 binding to PD-1 and CD80. Interim results from a phase I trial reported no colitis or pneumonitis of any grade, with several durable remissions, including NSCLC patients^56^. An ongoing phase I dose-escalation study (NCT01693562) of MEDI-4736 in 26 patients, 4 partial responses (3 in patients with NSCLC and 1 with melanoma) were observed and 5 additional patients exhibited lesser degrees of tumor shrinkage. The disease control rate at 12 weeks was 46%^57^. Expansion cohorts was opened in Sep 2013, 10 mg/kg q2w dose. 151 patients was enrolled so far in the expansion cohorts, tumor shrinkage was reported as early as the first assessment at 6 weeks and among the 13 patients with NSCLC, responses were sustained at 10 or more to 14.9 or more months^58^. In the NSCLC expansion cohort, the response rate was 16% in 58 evaluable patients and the disease control rate at 12 weeks was 35% with responses seen in all histologic subtypes as well as in a smaller proportion of PD-L1- tumors.

On the basis of the favorable toxicity profile and promising activity in a heavily pretreated NSCLC population, a global Phase III placebo controlled trial using the 10 mg/kg biweekly dose has been launched in Stage III patients who have not progressed following chemo-radiation (NCT02125461). The primary outcome measures are overall survival and progression-free survival.

#### AMP-224

AMP-224 was a B7-DC-Fc fusion protein which can block the PD-1 receptor competitively^59^. Some NSCLC patients were included in a first-in-man phase I trial of this fusion protein drug. A dose-dependent reduction in PD-1-high TILs was observed at 4 hours and 2 weeks after drug administration^60^.

## The combination therapy

A variety of approaches for combining PD-1/PD-L1 pathway inhibitors with other therapeutic methods have been explored over the past few years in an effort to offer more feasible therapeutic options for clinic to improve treatment outcomes. Approaches have included combinations with other immune checkpoint inhibitors, immunostimulatory cytokines (e.g. IFN-y) cytotoxic chemotherapy, platinum-based chemotherapy, radiotherapy, anti-angiogenic inhibitors, tumor vaccine and small-molecule molecularly targeted therapies many with promising results^61^,^62^. Studies indicated that PD-1/PD-L1 pathway inhibitors were most effective when combined with treatments that activating the immune system^63^.

Preclinical evidence exists for the complementary roles of CTLA-4 and PD-1 in regulating adaptive immunity, and this provides rationale for combining drugs targeting these pathways. In a Phase I study in 46 chemotherapy-naive patients with NSCLC, four cohorts of patients received ipilimumab (3 mg/kg) plus nivolumab for four cycles followed by nivolumab 3 mg/kg intravenously every 2 weeks. The ORR was 22% and did not correlate with PD-L1 status^64^.

In another Phase I study, 56 patients with advanced NSCLC were assigned based on histology to four cohorts to receive nivolumab (5–10 mg/kg) intravenously every 3 weeks plus one of four concurrent standard “platinum doublet” chemotherapy regimens. No dose de-escalation was required for dose-limiting toxicity. The ORR was 33–50% across arms and the 1-year OS rates were promising at 59–87%^65^.

At present, there are many other clinical trials of combination therapy are ongoing. A second stage of phase I trial testing about the combination of MEDI4736 with tremelimumab is also enrolling (NCT01975831). Other combinations regimens being tested include nivolumab in patients with NSCLC with chemotherapy, bevacizumab, erlotinib or ipilimumab (NCT01454102), and in advanced solid tumors with IL-21 (NCT01629758). Ongoing combination therapy trials with pembrolizumab include co-administration with cisplatin + pemetrexed or carboplatin + paclitaxel in NSCLC (NCT01840679). In addition, MPDL3280A is being studied in combination with cytotoxic chemotherapy in patients with NSCLC. Researches in regard to the therapy effectivity of combining PD-1/PD-L1 pathway inhibitors with IFN-g, cancer vaccine, soluble CD80 are ongoing or have got results in animal models and other tumor subtypes^66^.

Current research on the combination therapy between PD-1/PD-L1 inhibitors and radiotherapy primarily focuses on animal experimentation (tumor-bearing mice). In a study of glioblastoma multiforme (GBM), investigators tested the combination of anti-PD-1 antibody with stereotactic radiotherapy in a mouse orthotopic GBM model. The results demonstrated that combination-therapy can significantly prolong the survival compared with either modality alone. Long-term survival was seen only in the combination-therapy group, with some mice alive more than 180 days^67^.

In another study of preclinical melanoma and renal cell carcinoma mouse models, scientists found that PD-1 wild-type mice can recapitulate the radiotherapy-induced antitumor responses observed in PD-1 knockout mice after they received anti-PD-1 body therapy, thus prolonging their survivals. The combination of anti-PD-1 therapy and radiotherapy can also elicit the reduction in size of non-irradiated, secondary tumors outside the SABR radiation field (abscopal effect)^68^.

The feasibility of combination therapy was that radiotherapy maybe had an effect on up-regulation of PD-L1 on tumor cells^69^.

### Safety of drugs

The PD-1/PD-L1 inhibitors are considered to have a good safety, little toxicity and relatively well tolerated therapeutic method. In different trials (mentioned in this review), the incidence rate of immune-related adverse events (irAEs) exist large difference, but mainly focus on grade 1, 2, such as: fatigue, rash, pruritus, diarrhea, drug-related pneumonitis, vitiligo, colitis, hepatitis, hypophysitis, thyroiditis and vomit. However, there are still some reports which pointed out that a certain proportion of grade 3 or 4 irAEs occured. In a clinical trail involving a total of 296 patients, no maximum tolerated dose was defined, Grade 3 or 4 irAEs occurred in 14% of patients; three deaths resulted from pulmonary toxicity. In a clinical trial with combination therapy, the patients were followed up at least 4 months, grade 3-4 irAEs occurred in 22 of 46 (48%) and led to discontinuation in 16patients^63^. Three treatment-related deaths were due to respiratory failure, bronchopulmonary hemorrhage and toxic epidermal necrolysis. In another clinical trail, grade 3 or 4 treatment-related AEs were reported in 45% including pneumonitis, fatigue and acute renal failure^68^. The precise signaling pathways of these irAEs were still unclear. With careful vigilance, pneumonitis can often be controlled early with administration of corticosteroid.

## Prospects and problems

### Be a biomarker of prognosis

The PD-L1 expression has been shown to correlate with positive responses to PD-1/PD-L1 pathway inhibition in clinical studies and with poor prognosis in many cancers including NSCLC^45^,^46^. Available early data indicate that PD-L1 expression in tumors as a possible predictive biomarker of response to anti-PD-1/PD-L1 drugs. For example, in an interim phase I clinical trail, pretreatment tumor PD-L1 expression by immunohistochemistry (IHC) was considered as a statistically significant predictor of response. In evaluable archival samples, 6 of 9 PD-L1+ patients had responses compared with 1 of 24 PD-L1– patients^50^.

However, there are still some controversies for this viewpoint. PD-L1 is an inducible molecule and tumors are frequently heterogenous. Therefore, the threshold of PD-L1 expression positive or overexpression is different in different trials. In addition, even for the same patients, if we observe different region cells, the threshold is different too. For example, discordance between primary tumor and metastases for PD-L1 positivity in both directions has been observed in kidney cancer in 15% (5 of 34) of patients^70^. Finally, PD-L1 expression appears to be less relevant in combination immunotherapy regimens. For example, the response to the combination of ipilimumab and nivolumab mentioned above appeared to be unrelated to PD-L1 expression.

So, the role of tumor expression of PD-L1 must be further elucidated. Further clinical studies should address these associations.

### Problems currently and the future research orientation

Although much is known about the PD-1/PD-L1 pathway in tumor immunity, many problems remain unexplored. For example: According to different patients, how to choose an appropriate therapic approach, PD-1/PD-L1 inhibitors or chemotherapy or combination therapy or others and whether there are different curative effect between PD-1/PD-L1 inhibitors and other therapies and does the differences make sense? Whether PD-1/PD-L1 inhibitors can be used for the first-line? Does blockade of the PD-1/PD-L1 pathway result in activation of only tumor-reactive T cells or also T cells that are auto-reactive to non-tumor antigens and that could cause undesirable autoimmunity? Whether there are any other factors impact research results about the biological markers, such as: PD-L2, or other gene mutation or expressed proteins, whether could predict prognosis by two factors or more? Thus, additional basic immunology studies and larger samples of clinical trials are essential.

## Conclusion

The research of cancer immunotherapy provides a new wide space for cancer treatment (including NSCLC), and compared with other therapeutic method, immunotherapy has its unique advantages, such as: relative safety, effectivity, less and low grade side effect and so on. Especially with the discovery and continued in-depth study of PD-1/PD-L1 pathway in the immune regulation mechanism, many significative conclusions were reported. Data from many clinical trails suggest that some patients with NSCLC have been benefited from the drugs of anti-PD-1 and anti-PD-L1 already. However, summarized what have been discussed above, only a small fraction of patients benefit from PD-1 or PD-L1 inhibitors treatment. But with the continuous studies on biomarker and combined treatment in PD-1/PD-L1 pathway, new research progress will be acquired as well. We will make significant progress on treatment and in control of NSCLC.

## Additional Information

**How to cite this article**: He, J. *et al.* Development of PD-1/PD-L1 Pathway in Tumor Immune Microenvironment and Treatment for Non-Small Cell Lung Cancer. *Sci. Rep.* **5**, 13110; doi: 10.1038/srep13110 (2015).
